# Availability and Utilization of WHO Lifesaving Medicines for Children under Five in Public Health Facilities of the Jimma Zone, South West Ethiopia: A Cross-Sectional Survey

**DOI:** 10.1155/2020/3505672

**Published:** 2020-05-31

**Authors:** Tidenek Mulugeta Tujo, Tadesse Gudeta Gurmu

**Affiliations:** School of Pharmacy, Institute of Health, Jimma University, Ethiopia

## Abstract

**Background:**

The increased morbidity and mortality rates in children under five in developing countries are mostly attributed to poor availability and failure of prescribing lifesaving medicines. This study was aimed at evaluating the availability and utilization of the WHO-recommended priority lifesaving medicines for children under five in public health facilities.

**Method:**

A cross-sectional survey complemented with a qualitative method was conducted in 14 health centers and four hospitals in the Jimma Zone, Ethiopia. In the facilities, we assessed the availability within the last half year and on the day of the visit. Utilization of the medicines was assessed through a review of patient records of the last one year. Twelve in-depth interviews were carried out to collect the qualitative data, and the analysis was executed using thematic analysis.

**Results:**

For treatment of pneumonia, amoxicillin dispersible tablets and gentamycin injection were available in 94.4% of the facilities. For treatment of malaria, artemether/lumefantrine was available in 61.1% of the facilities. For pain management, paracetamol tablets were available in 94.4% of the facilities. AZT+3TC+NEV for HIV/AIDS management was available in all facilities. At least one essential medicine was out of stock in the past six months with the average duration of 33.6 days in health centers and 28.25 days in hospitals. Oral rehydration salt and zinc (84.7%) and AZT+3TC+NEV (100%) had better utilization. However, for almost all cases, other nonpriority medicines were highly prescribed. Lack of administrative commitment, supply of near expiry products, complexity of diseases, and lack of customized child formulations were among the challenges of availability and utilization of those medicines.

**Conclusions:**

The overall availability of lifesaving medicines on the day of the visit was fairly good but with poor utilization in almost all facilities. Some products were not available for considerable length of time in the past six months.

## 1. Background

According to UNICEF's (United Nations International Children's Emergency Fund) report, globally, there is notable progress in child survival observed in the past few decades. Millions of children have better survival chances than those in 1990. One in twenty-six children died before reaching the age of five in 2017, compared to one in eleven in 1990. Despite the global progress in reducing child mortality, an estimated 5.4 million children under the age of five died in 2017. Roughly half of those deaths occurred in sub-Saharan Africa [1].

The World Health Organization (WHO) reported that the majority of the morbidities in children under five are preventable or treatable if lifesaving medicines are available and consistently utilized [2]. To tackle this problem, the WHO, United Nations Population Fund (UNFPA), and UNICEF have jointly developed a list of priority medicines. The list identified malaria, pneumonia, sepsis, pain, diarrhea, HIV, and vitamin A deficiency as the leading causes of morbidity in children under five [3].

Making lifesaving medicines available in different facilities contributes a lot to reducing the newborn and child morbidity and mortality [4]. However, availability of a product solely will not be a guarantee to achieve the target of the products. In addition to ensuring availability, it is also important to assess and verify whether the medications are being prescribed or not [5].

Medicines are among the most effective and widely used therapeutic interventions in health care today. Despite this important role of medicines, it is difficult to realize their role without knowing the health practitioners' habit of prescribing the recommended medicines. Information on this is the anchor of any system to produce the desired effects [6].

Nonavailability of priority medicines pushes health care providers to prescribe alternative medicines. Such an approach may finally end up with treatment failure and associated increased consultation rates [7]. On the other hand, though the products are available physically, failure to prescribe them particularly reduces the quality of medical care and leads to the wastage of resources [8].

According to the report of the United Nations (UN) commission, two of the issues facing developing countries include insufficient supplies of health commodities and associated utilization problems [9].

A cross-sectional survey conducted in 32 lower-level public facilities in Jinja District of Uganda showed that the availability and utilization of priority lifesaving medicines for pneumonia and malaria are low. However, the priority medicines for diarrhea and sepsis were available and highly prescribed by the health workers [10]. Similar lower availability of children's essential medicine was reported in Western Ethiopia and Guatemala [11, 12].

The majority of previous studies focused mostly on the evaluation of essential medicine availability for a general pediatric population. However, studies specifically addressing medicines for children under five are scarce. On top of that, little is known about the utilization of those medicines. Even the available limited studies relied on the prescriber's self-report to evaluate the medicine utilization practice. Therefore, the aim of the present study was to evaluate the availability and utilization of WHO lifesaving medicines for children under five.

## 2. Methods

### 2.1. Study Design and Setting

A cross-sectional survey complemented with a qualitative method was conducted in selected public health facilities in the Jimma Zone from March 5 to April 9, 2018. The Jimma Zone is geographically located to the southwest of Ethiopia, 350 km away from the capital city of the country, Addis Ababa. It is administratively subdivided into 18 districts, namely, Agaro Town, Chora Botor, Dedo, Gera, Gomma, Guma, Kersa, Limmu Kosa, Limmu Sakka, Mana, Omo Nada, Seka Chekorsa, Setema, Shebe Senbo, Sigmo, Sokoru, Tiro Afeta, and Jimma Town. Currently, the zone has a total of 630 public health facilities, i.e., seven hospitals, 111 health centers, and 512 health posts giving service to the community. The Ethiopian pharmaceutical supply agency Jimma hub is the main source of medicines for these public health facilities. Three thousand and nine hundred ninety health professionals of different fields had been serving in those facilities [13].

### 2.2. Source Population and Study Units

The source population of the current study was all health facilities, health workers, logistics documents, and medicines. The study units were WHO-recommended priority lifesaving medicines, selected public hospitals, and health centers. The study observations include bin cards, patient cards, and selected health professionals.

### 2.3. Inclusion and Exclusion Criteria

#### 2.3.1. Inclusion Criteria

To capture a complete image of a product stockout and the associated challenges, facilities which provided services for more than a year (i.e., just from the day of the visit) were included in the study. Experience of greater than six months was the inclusion criteria for the study participants.

#### 2.3.2. Exclusion Criteria

Since ART (Antiretroviral Therapy) drugs were in the list of WHO lifesaving medicines, facilities which do not provide ART service were excluded. According to the Ethiopian health structure, health posts are under the umbrella of health centers and authorized to provide only minor services. Moreover, to maintain the consistency of data, sepsis illness was excluded from the study as it is not managed at a health center level in Ethiopia.

### 2.4. Sample Size Determination and Sampling Procedures

**Figure 1:**
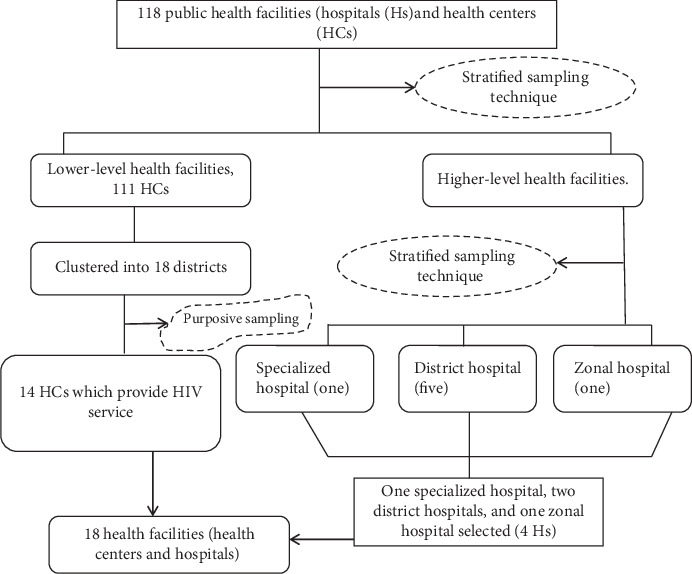
Flow diagram showing the health facility selection procedure.

To determine the number of facilities, the Logistics Indicators Assessment Tool (LIAT) was used. It recommends taking at least 15% of the total facilities [14]. The sample size for the current study was calculated from the total number of hospitals and health centers by taking the inclusion and exclusion criteria into account. Accordingly, the total number of health centers and hospitals in the zone gives 118. Thus, based on the LIAT guideline, the sample size became 18 (i.e., 15% of 118 = 17.7≅18). As the facilities differ in terms of setting and scope of services, we stratified them as lower-level facilities (health centers) and higher-level facilities (hospitals). The hospitals were further stratified as a specialized hospital, district hospital, and zonal hospital. Accordingly, two district hospitals, one zonal hospital, and one specialized hospital were included. Three district hospitals were excluded since they were newly established just less than one year prior to the study. The health centers were clustered based on districts. As per the Ethiopian health care system, only one health center per district provides HIV treatment service. Therefore, 14 health centers which manage antiretroviral drugs were purposively chosen from the districts from which hospitals were not selected. The sampling procedure for the facilities is depicted in Figure 1.

Regarding product selection, nineteen WHO-recommended priority lifesaving medicines for pneumonia, malaria, diarrhea, HIV, pain management, and vitamin A deficiency were considered. For pneumonia, amoxicillin dispersible tablets, ampicillin injection, gentamycin injection, and ceftriaxone injection were included. For malaria, artemether/lumefantrine, artesunate injection, and rectal artesunate were included. For diarrhea, ORS and zinc were included. For pain management, paracetamol dispersible tablets and morphine were considered. For vitamin A deficiency, vitamin A was considered [3]. For HIV management, the WHO list does not specify medications; rather, it recommends referring to country-specific lists. Therefore, the Ethiopian HIV treatment guideline was consulted to incorporate medications [15]. Accordingly, for HIV, AZT+3TC+NEV, AZT+3TC, NVP, ABC+3TC, 3TC, LPV/r, and EVF were included.

Document reviews were also performed to gather essential data. We reviewed bin cards to evaluate stockout rates in the past six months and patient cards to determine the utilization of lifesaving medicines. The number of bin cards was based on the amount of medicines and the total number of facilities included in the study. Accordingly, one bin card for each product was expected from each study facility; unfortunately, bin cards for two products, morphine from health centers and artesunate suppository from both types of facility, could not be accessed. In Ethiopia, health centers are not allowed to manage morphine. Additionally, artesunate suppository was totally unavailable in the facilities for the last three years. Therefore, a total of 310 bin cards (238 from health centers and 72 from hospitals) were randomly reviewed. The number of patient cards was calculated using the single population proportion formula and adjusted with a finite correction factor.

(1) $$\begin{array}{c} n=\frac{{\left(z_{\alpha 1_{/2}}\right)}^{2}*P\left(1-P\right)}{m^{2}}=384, \end{array}$$

where *n* is the sample size from the formula, *z*_*α*1/_2__ is the value of confidence, the level of 95% is 1.96, *p* is the proportion of patient cards (50%), and *m* is the margin of error (at 5% *m* = 0.05).

However, before computing the final sample size, the authors obtained data regarding the incidences of the WHO priority diseases for children under five within the past one year (prior to the study) from the Jimma zonal health bureau. Consequently, about 5745 encounters with the said problems were identified. Thus, since the frame of sampling (5745) was less than 10,000, the final sample size was achieved by adjusting the sample obtained from general formula (384) using a finite correction factor.

(2) $$\begin{array}{c} n^{\prime }=\frac{n}{\left[1+\left(\left(n-1\right)/N\right)\right]}=360\;patient\;cards, \end{array}$$

where *n*′ is the adjusted final sample size, *n* is the sample size obtained from the general formula, and *N* is the sampling frame (5745).

The last sample size was then distributed based on the number of cases per facility. However, as sepsis is not managed at a health center level, only five of the six WHO priority cases of children under five were used. As a result, four cards per case per facility were drawn until the sample size was achieved. Card numbers from the register book have been used to obtain the required patient document. Patient cards of the last one year were considered for sampling.

The storekeepers of all the study facilities took part in the quantitative study to provide data on factors affecting product availability.

### 2.5. Data Collection Procedures

Data regarding availability were collected using questionnaires and checklists adapted from the USAID deliver guideline [14]. And the tools for assessing the utilization practices were developed by the investigators after reviewing related literatures. Two types of questionnaires were used: structured and semistructured questionnaires. The structured self-administered questionnaires were distributed to the storekeepers to collect data on factors affecting availability of lifesaving medicines such as logistics system-related factors. The semistructured questionnaires were used to collect qualitative data during the in-depth interviews. The checklists had three parts; the first part contained questions regarding the availability of medicines on the day of the visit, the second part contained questions used to collect data on availability in the past six months, and the third part was designed to collect data on the utilization of lifesaving medicines.

Physical verification conducted to determine availability on the day of the visit and percentage availability was computed as the proportion of facilities with the WHO-recommended priority lifesaving medicines. Bin cards of the past six months were randomly picked and reviewed for each product to determine stockouts. The sampled patient cards of one year were checked to determine the utilization practice. The quantitative data were collected by three trained druggists under the supervision of investigators. Half-hour training was given to the data collectors regarding the data collection procedures and ethical issues.

For the qualitative part, twelve in-depth face-to-face interviews were conducted with selected key informants including medical directors, pharmacy unit heads, store personnel, health officers, nurses, and medical doctors. For the purpose of maintaining consistency, the interviews were conducted by the principal investigator. The data collection was an iterative process. The majority of the interviews were conducted in the respondents' office/workplace, whilst the rest were carried out outside the office for the convenience of the respondents. Each interview lasted between 20 and 30 minutes. An open-ended interview guide was used to assess the perception of the key informants. For the purpose of convenience, the interviews were conducted in Amharic language and tape-recorded. The investigators then transcribed the records into English, and an expert from Jimma University verified the transcription.

### 2.6. Data Analysis

The study employed Statistical Package for the Social Sciences (SPSS) version 20 to analyze the quantitative data. A descriptive statistical analysis was used to determine frequency counts, averages, and percentages. To define statistical associations between the dependent and independent variables, the chi-square test was used. A *P* value less than 0.05 was used to determine a statistical significance. The results were presented using tables and texts according to the nature of the data. The utilization was calculated as the percentage of individual medicines prescribed for specific diseases stipulated in the patient cards.

The data analysis for the qualitative study was done manually using a content analysis technique. Data were coded in a word document using a table, and then, the codes were used to search for themes. Finally, reports were produced and triangulation of the findings with quantitative results was carried out, and some of the responses were quoted.

### 2.7. Operational Definitions

1. *Utilization* refers to prescribing the required WHO-recommended lifesaving medicines for the management of a specific disease in their recommended dose and formulation
2. *The last six months* refers to six months prior to the study period
3. *The last one year* refers to one year prior to the study period
4. *Supervision* is a process that involves a manager meeting regularly and interacting with worker(s) to review their work
5. *Products* refer to the ones interchangeably used with WHO-recommended priority lifesaving medicines
6. *Patient document* refers to the patient card

The following ranges were used for describing availability [16]:

- High means >80%
- Fairly high means 50% to 80%
- Low means 30%–49%
- Very low means <30
- However, for the purpose of the chi-square test, we merged them into high availability (>80%) and low availability (≤80%)

## 3. Results

### 3.1. Availability and Utilization of the WHO-Recommended Priority Lifesaving Medicines for Children under Five

The average availability of WHO priority medicines was 63.9% for the health centers and 82.9% for the hospitals. As per WHO classification, availability was fairly high for the health centers and high for the hospitals.

#### 3.1.1. Management of Pneumonia

Almost all medicines for pneumonia treatment were available in the hospitals and in more than 80% of the health centers. Specifically, amoxicillin dispersible tablets and gentamycin injection were available in 94.4% of the study facilities. However, only 20% ceftriaxone injection and 36.1% amoxicillin dispersible tablets were prescribed for the case.

#### 3.1.2. Malaria (*Plasmodium falciparum*) Case

Artemether/lumefantrine (61.1%) and artesunate injection (50%) were available for the management of malaria. The former was available in 75% of the hospitals and 57.1% of the health centers. Twenty-four percent of children diagnosed with a malaria case were prescribed with artemether/lumefantrine.

#### 3.1.3. Diarrhea Management

Around 94% of diarrheal cases in the study facilities were managed using oral rehydration salt and zinc. ORS was available in 85.7% of the health centers and in all of the hospitals. Very few cases (15%) were treated with antihelminthics.

#### 3.1.4. Pain Management

Paracetamol tablets in children under five were available in 94% of the facilities. However, only 6.9% of patients were prescribed with the paracetamol tablet. More than half of the patients (55%) in the study facilities received other nonpriority medicines like paracetamol suppository and syrup. It had also only 2.8% utilization in the hospitals.

#### 3.1.5. HIV/AIDS Therapy

All health centers and hospitals used the AZT+3TC+NEV combination for HIV/AIDS treatment in children under five. The combination was also available in all health facilities. Despite their availability, the other WHO priority medicines were not used in the facilities as the patients were on the first-line ART (Table 1).

### 3.2. Stockout Rates for WHO Lifesaving Medicines

All of the hospitals and health centers faced a stockout of one or more medicines in the past six months prior to the study. The most frequent stocked out item in health centers was 3TC (10 mg/ml) with a mean frequency of 1.3 times. In hospitals, lopinavir/ritonavir was the most frequently stocked out item with a mean frequency of 2.3 times. Further, the duration of the stockout was longer for lopinavir/ritonavir (129 days) and lamivudine solution (112 days) in health centers. In hospitals, artesunate injection was out of stock for about 10 days. The mean total stockout duration was 33.6 days in health centers, and it was 28.3 days in hospitals (Table 2).

### 3.3. Product Availability-Related Factors

Regarding availability, the storekeepers mentioned influencing factors like order change at a resupply point (13, 72.2%); the frequency of facility supervision from the higher management (18, 100%, monthly); the frequency of sending a request form to a supplier, e.g., monthly (5, 27.8%), bimonthly (10, 16.7%), quarterly (33.3%), or after three months (4, 22.2%); the stockout at a supplier point (12, 66.7%); and means of transportation cost coverage like using own vehicle (22.2%), rental (8, 44.4%), or supplier delivery (2, 11.1%).

### 3.4. Inferential Statistical Analysis

From the chi-square test, a statistically significant association was observed between availability and frequency of supervision (*P* = 0.003), order change at a resupply point (*P* = 0.017), means of transportation cost coverage (*P* = 0.018), and stockout at a resupply point (*P* = 0.001) (Table 3).

### 3.5. Qualitative Results

#### 3.5.1. Challenges Associated with Availability

From the in-depth interviews, two striking themes related to availability of WHO-recommended lifesaving medicines were identified, i.e., management support- and supply system-related challenges.

*(1) Management Support-Related Challenges*. Regarding the management system, the majority of the facilities experienced bureaucratic, nonflexible financial procedures and poor commitment of administrators at different levels. One of the interviewees explained that

“Most of the time, because of non-responsiveness and rigidity of the facility management, it is challenging to procure items from private sellers when the products get stock out from the government supplier. Especially, the procedural elongation and lack of flexibility of the finance system is a big problem. It is annoying to see the service interrupted because of the negligence of the management. Their quick response could save children's lives and also protects parents from unnecessary and unexpected expenses.”

The other problem was regarding human resource. Shortage of skilled human power tends to increase the workload and ultimately affects the performance of an organization as a whole. The majority of the study facilities particularly, health centers, had scarcity of pharmacy professionals. As a result, handling the different logistics activities became difficult and led to stockout of the lifesaving medicines. One storeman reported that

“I have multiple responsibilities in my facility including dispensing drugs serving as a pharmacy head and managing the store. Because of the workload, I could not regularly review records and physically verify medicines to determine how much to request and when to place orders. This can be one of the reasons for a frequent stock out of medicines. The shortage of human resource is usually because of insufficient budget.”

*(2) Supply System-Related Challenges*. The interviewees reported that the communication gaps between facilities and suppliers, particularly the Ethiopian Pharmaceuticals Supply Agency (EPSA), affect the sustainable availability of medicines. Besides the communication gaps, the supplier (EPSA) sometimes intentionally pushes medicines without considering the facilities' need. Even most of the medicines were with short expiry dates. One of the pharmacy heads stated that

“Most often, despite maximum efforts exerted in preparing and sending quality reports to the supplier, the problems remain unsolved. The pharmaceutical supply agency of the country supplies the excess items of its warehouse rather than responding to actual requests. The most important items which are relevant to our facilities' program are usually stock out.”

The other problem was in relation to the transportation system. Most health facilities, particularly the rural health centers, had transportation problems. Some of these facilities therefore use motor bikes and office vehicles to deliver the items. They also plan small amounts less than their actual demand. One medical director from a health center emphasized the issue as follows:

“Even though opportunities are there to borrow office vehicles, they usually have additional duties which enforced us to use rental vehicles. On top of that, it is impossible to find vehicles, which are appropriate for pharmaceutical transportation. The governmental supply agency, which is our main supplier, does not provide transportation service regularly. This increases our transportation costs and prevents us from purchasing the required amounts.”

#### 3.5.2. Medicine Use-Related Challenges

The problems like unavailability of medicines, absence of child-friendly formulations, changes in treatment protocols, and the associated information gaps were some of the concerns raised in relation to the utilization of WHO lifesaving medicines. For the ease of reporting, the issues were categorized into guideline-related and product-related challenges.

*(1) Guideline-Related Challenges*. From the interview of prescribers, one of the causes for underutilization of the WHO-recommended lifesaving medicines was a mismatch between WHO and Ethiopian treatment protocols. For instance, ampicillin and gentamycin injections for pneumonia and morphine for pain management are not drugs of choice in the Ethiopian treatment guideline. Some of the interviewees reported that the treatment protocols for some cases were frequently changing and the practitioners seldom get the updates timely. Plus, the newly recommended medicines might not be available in the facility. One participant pointed out that

“We typically experience changes in treatment regimens to use the WHO recommended medicines. Even if the new drugs are from WHO priority medicines, we may not have the product in our stock. Since treatment protocols get changed at national level by federal ministry of health, the facilities and practitioners are usually informed late. Therefore, we continue procuring and using the former drugs.”

Lack of access to up-to-date and reliable drug information also affected the prescribing practice. The majority of the clinicians, especially junior practitioners, prescribed based on trends. Others mentioned medical textbooks as the only accessible source of drug information. One of the prescribers stated that

“I usually prescribe based on a disease management trend I learned during my training in the university. What I can easily access are the medical textbooks, which are usually outdated. Our facility does not have a library or any drug information center to access updated information. I believe access to information could improve prescribing practice.”

The other reason was regarding disease severity classifications. The WHO guideline does not categorize the illnesses based on severity. The WHO recommendations often work for uncomplicated cases. However, the real scenario in the facility is usually different. There were clinical complexities, which forced the prescribers to follow advanced clinical approaches. One medical doctor reported that

“It is appreciable to use treatment guidelines and I also try to follow them. However, some treatments need individualization based on the patient's functional ability, need and severity of the disease. The recommendations like that of WHO lacks detail and flexibility for such cases. The clinical recommendations should not place an additional burden on children who are already suffering from painful diseases.”

*(2) Product-Related Challenges*. In the majority of facilities, the resupply of essential medicines did not consider child-friendly formulations. As a result, clinicians faced difficulty in adhering to treatment guidelines; most of them were calculated from an adult dose. One key informant highlighted that

“In my experience as a health officer, sometimes the recommended product may not be available in the stock in the recommended formulation. Thus, I am enforced to prescribe alternative medicines and alternative dosage forms out of the suggestion of WHO e.g.by crashing tablets. I do this to save families from spending extra cost in purchasing from private pharmacies. However, such practice may not be safe and effective for babies, it is also difficult for them to swallow a processed medicine like a crashed tablet because of its unpalatable tastes.”

The other product-related challenge was regarding the WHO formulations for children under five. The WHO guideline recommends an oral solid dosage form as the first line over the other formulations for children under five. However, babies prefer liquid oral dosage formulations such as syrup. Caregivers also face difficulties in administering a solid dosage form. One medical doctor reported that

“Families of the children always challenge us to prescribe a liquid dosage form. They complain that children spit out the tablets. Syrup gives more precise and clearly calculated doses for the child. Although the WHO does not recommend, we commonly prescribe the syrups so that the caregivers feel comfortable to administer the formulation. By doing this, we expect adherence to medications would improve.”

## 4. Discussion

### 4.1. Availability of WHO-Recommended Lifesaving Medicines for Children under Five

The finding of the current study revealed that the average availability of priority medicines for children under five lay within a fairly high to high range, which was 63.9% in health centers and 82.9% in hospitals. This is in line with the study conducted in Mongolia [17]. However, it deviates from the finding of a study conducted in Guatemala (25%) [12]. This might be due to the difference in the type of facilities included in the Guatemalan study, where private facilities were also considered.

There was a variation in the availability of specific drugs, and it was difficult to find some formulations that are preferable for use in children. Artesunate suppository, which was not available in any of the facilities, can be a good example. Similar findings were obtained from a study conducted in the western part of Ethiopia [11]. From the in-depth interviews, the majority of the facilities did not give attention for pediatric medicines. They believe that an adult dosage regimen could be adjusted for children. Thus, they rarely update their procurement list in order to incorporate child formulation though different appropriate dosage forms are required for kids. The key informants also stated that shortage of skilled human power could be one of the main reasons for mass procurement of products. The available staffs were busy to review records and identify items based on the age group categories.

The availability of AZT+3TC+NEV, ORS, zinc, amoxicillin dispersible tablets, vitamin A, gentamycin, and ceftriaxone was high in both hospitals and health centers. In contrast to the present study, a study conducted in Uganda reported low availability of vitamin A and pediatric antiretroviral drugs [12]. This disparity might be attributable to the consumption difference of those medicines following the difference in the disease pattern of the two countries. Similarly, the key informants also mentioned that the supplier's failure to avail the most important items in the required quantity could also make a difference. What the facilities actually demand and what the vendor supplies are usually different.

All of the facilities faced a stockout of one or more medicines in the past six months. The most frequent stocked out item in the health centers was 3TC (10 mg/ml) with a mean frequency of 1.3. Lopinavir/ritonavir combination was the most frequently stocked out product in the hospitals with a mean frequency of 2.3 times. Further, stockout duration was longer for lopinavir/ritonavir (129 days) and lamivudine solution (112 days) in health centers. The mean stockout duration was 33.6 days in health centers and 28.3 days in hospitals. However, products in the current study were out of stock for shorter periods compared to those in the study conducted in Indian districts wherein the average stockout duration was 66.1 days [18]. The possible reason could be the difference in the length of time under consideration to review logistics records (eight versus six months) for the assessment of stockout status. The qualitative result implicated that such product stockouts are attributed to lack of commitment of the management and the supply of near expiry products. Especially, the lengthy and nonflexible financial process during purchasing of items worsens the problem. Additionally, transportation problems and order changes at a resupply point significantly affected availability.

### 4.2. Utilization of WHO-Recommended Lifesaving Medicines for Children under Five

The majority of children in the current study had received less than 50% of the recommended antibiotics for the treatment of pneumonia. Amoxicillin dispersible tablets (36.1%) and ceftriaxone injection (27.8%) were relatively better prescribed for the case. The present findings are almost similar to a report from the integrated Global Action Plan for Pneumonia and Diarrhoea (GAPPD) [19] but lower than the findings from Southern Malawi where 68.2% of children received the recommended antibiotics [20]. The discrepancy might be due to the difference in the contents of the two countries' treatment guidelines in aligning with the WHO treatment protocols. The evidence from the in-depth interview also showed that the treatment guideline of Ethiopia is somehow different from that of the World Health Organization.

Regarding diarrhea management, both WHO and Ethiopian treatment guidelines recommend oral rehydration salt (ORS) and currently the combination of ORS and zinc as the best treatment options. In the present study, around 85% of children received ORS and zinc for the management of diarrheal cases within the past one year. The facilities of the current study showed better adherence to the WHO guideline in prescribing ORS and zinc, compared to an assessment conducted by Management Sciences for Health (MSH) in 2012 where only 31% ORS and 1.5% zinc were prescribed. The difference might be due to the exhaustive promotion and vigorous awareness creation done nationally through different means including broadcasting media [21].

In relation to the utilization of antimalarial medicines, 33.3% of children in the current study received artemether/lumefantrine and 27.8% of them received artesunate injection. The findings are much higher than a report from Uganda where none of the prescribers used artemether/lumefantrine and artesunate injection [10]. The discrepancy might be due to a recall bias emanating from prescribers' self-report in Uganda. In the current study, we reviewed patient cards to determine the level of utilization. Even though artesunate suppository is a suitable dosage form for accurate administration, the study facilities could not have access to the product to be prescribed for their patients.

Regarding pain management, the WHO guideline recommends paracetamol or morphine in flexible oral solid dosage formulation for children under five. It also emphasized that a flexible oral solid dosage form is the most preferable. However, in the current study, only 6.95% of the children had received a paracetamol tablet. During the in-depth interview, the key informants reported that a solid dosage form is not convenient for administration in children. That is why the percentage of paracetamol tablet utilization was very low. Children spit out tablets. They rather prefer taking palatable liquid oral dosage formulations such as syrup.

According to the finding of the current study, all of the children had received standard regimen first-line antiretroviral treatment. However, the key informants reported that sudden changes in clinical recommendations and the associated delay in information exchange between different levels were among the challenges they faced in their daily activities. Similar findings were obtained in a study conducted in Ugandan health facilities, where a change in the guideline of malaria management interfered the utilization pattern of antimalarial medications [22].

This study has potential limitation. World Health Organization defines drug utilization from the perspective of prescribers, dispensers, and patients. However, this study addressed from the prescribers view point only.

## 5. Conclusion

In general, the overall availability of WHO-recommended priority lifesaving medicines for children under five was high on the day of the visit. However, some lifesaving medicines were not available for longer duration in the past six months. As these medications are lifesaving, their absence even for a day means a lot. The contributing factors for their absence were less frequent supervision, order changes at a resupply point, lack of appropriate transport, and stockout from a resupply point. Key informants also reported that lack of commitment from the management and the elongated nonflexible financial process, low levels of staffing, and supply system-related challenges like resupply of near expiry and unwanted products were the major challenges. Furthermore, there was a great gap in the availability of child-appropriate formulation varieties. For some of the products, even though their availability is good, their utilization was low. On top of this, challenges like product unavailability, nonflexibility of WHO's recommendation, sudden recommendation changes, and information gaps were identified as major challenges of prescribing those medicines.

## Figures and Tables

**Table 1:** Availability and utilization of WHO-recommended priority lifesaving medicines for children under five in public health facilities of the Jimma Zone, May 2018.

Cases	WHO-recommended priority lifesaving medicines	Availability on the day of the visit		Aggregate availability, *N* = 18 (%)	Utilization		Aggregate utilization, *N*∗ = 72 (%)
		Health centers, *N* = 14 (%)	Hospitals, *N* = 4 (%)		Health centers, *N*∗ = 56 (%)	Hospital, *N*∗ = 16 (%)	
Pneumonia	Amoxicillin dispersible tablet	13 (92.9)	4 (100.0)	17 (94.4)	15 (26.8)	11 (68.8)	26 (36.1)
	Ampicillin injection	10 (71.4)	4 (100.0)	14 (77.8)	0 (0.0)	0 (0.0)	0 (0.0)
	Gentamycin injection	13 (92.9)	4 (100.0)	17 (94.4)	0 (0.0)	1 (6.3)	1 (1.4)
	Ceftriaxone injection	12 (85.7)	4 (100.0)	16 (88.9)	10 (17.8)	10 (62.5)	20 (27.8)
	Others^1^ (nonpriority)	NA	NA	NA	23 (41.1)	2 (12.5)	25 (34.7)

Malaria (*Plasmodium falciparum*)	Artemether/lumefantrine dispersible tablet	8 (57.1)	3 (75.0)	11 (61.1)	20 (35.7)	4 (25.0)	24 (33.3)
	Artesunate injection	7 (50.0)	2 (50.0)	9 (50.0)	16 (28.6)	4 (25.0)	20 (27.8)
	Rectal artesunate	0 (0.0)	0 (0.0)	0 (0.0)	0 (0.0)	0 (0.0)	0 (0.0)
	Others^2^ (nonpriority)	NA	NA	NA	22 (39.3)	2 (12.5)	24 (33.3)

Diarrhea	ORS	12 (85.7)	4 (100.0)	16 (88.9)	36 (64.3)	15 (93.8)	61 (84.7)
	Zinc	13 (92.9)	4 (100.0)	17 (94.4)	36 (64.3)	15 (93.8)	61 (84.7)
	Others^3^ (nonpriority)	NA	NA	NA	6 (10.7)	5 (32.2)	11 (15.3)

Pain management	Paracetamol dispersible tablet	14 (100.0)	3 (75.0)	17 (94.4)	2 (3.6)	3 (18.8)	5 (6.9)
	Morphine dispersible tablet	0 (0.0)	3 (75.0)	3 (16.7)	0 (0.0)	2 (12.5)	2 (2.8)
	Others^4^ (nonpriority)	NA	NA	NA	36 (64.3)	4 (25.0)	40 (55.6)

HIV/AIDS	AZT+3TC+NEV	14 (100.0)	4 (100.0)	18 (100.0)	56 (100.0)	16 (100.0)	72 (100.0)
	AZT+3TC	12 (85.7)	4 (100.0)	16 (88.9)	0 (0.0)	0 (0.0)	0 (0.0)
	NVP	13 (92.9)	4 (100.0)	17 (94.4)	0 (0.0)	0 (0.0)	0 (0.0)
	ABC+3TC	4 (28.6)	3 (75.0)	8 (44.4)	0 (0.0)	0 (0.0)	0 (0.0)
	3TC	2 (14.3)	4 (100.0)	6 (33.3)	0 (0.0)	0 (0.0)	0 (0.0)
	LPV/r	2 (14.3)	2 (50.0)	4 (22.2)	0 (0.0)	0 (0.0)	0 (0.0)
	EVF	10 (71.4)	3 (75.0)	13 (72.2)	0 (0.0)	0 (0.0)	0 (0.0)

Vitamin A deficiency	Vitamin A	11 (78.6)	4 (100.0)	15 (83.3)	56 (100.0)	16 (100.0)	72 (100.0)

*N*∗ = number of patient cards per case; *N* = number of facilities; NA = not applicable. ^1^Amoxicillin syrup and trimethoprim-sulfamethoxazole syrup. ^2^Chloroquine syrup. ^3^Anthelmintic. ^4^Paracetamol suppository and syrup.

**Table 2:** Stockout status for WHO-recommended priority lifesaving medicines for children under five in Jimma Zone health facilities, May 2018.

List of medicines	Health centers		Hospitals		Inclusion in NEDL (1 = yes, 2 = no)	
	Days of stockout for the past 6 months	Mean frequency of stockout	Days of stockout for the past 6 months	Mean frequency of stockout	Health center	Hospital
Amoxicillin dispersible tablet	7.0	1.0	0.0	0.0	1.0	1.0
Ampicillin injection	3.0	1.0	3.0	0.0	1.0	1.0
Ceftriaxone injection	10.0	1.1	3.0	1.0	1.0	1.0
ORS	5.0	1.0	0.0	0.0	1.0	1.0
Zinc dispersible tablet	5.0	1.0	0.0	0.0		
Artesunate injection	11.0	1.0	17.0	1.0	1.0	1.0
Artesunate rectal	NA	0.0	NA	0.0	1.0	1.0
Gentamycin injection	3.0	1.0	0.0	0.0	1.0	1.0
Artemether/lumefantrine dispersible tablet	18.0	1.0	11.0	1.0	1.0	1.0
Morphine dispersible tablet	NA	0.0	8.0	0.0	2.0	1.0
Paracetamol dispersible tablet	0.0	1.0	5.0	1.0	1.0	1.0
AZT+3TC+NEV (60/30/50 mg)	0.0	1.1	0.0	0.0	1.0	1.0
AZT+3TC (60/30 mg)	0.0	1.0	2.0	1.0	1.0	1.0
NVP (10 mg/ml)	11.0	1.1	0.0	0.0	1.0	1.0
ABC+3TC (60/30 mg)	77.0	1.1	6.0	1.1	1.0	1.0
3TC (10 mg/ml)	112.0	1.3	2.0	1.8	1.0	1.0
LPV/r (80 mg/20 mg)	129.0	1.0	8.0	2.3	1.0	1.0
EVF (50 mg)	8.0	1.0.0	4.0	1.0	1.0	1.0
Vitamin A	7.0	1	2.0	1.0	1.0	1.0
Total	471.0	—	113.0	—		
Average	33.6		28.3			

NA = not applicable; NEDL = national essential drug list.

**Table 3:** Chi-square test for factors associated with the availability of priority lifesaving medicines for children under five in Jimma Zone health facilities, May 2018.

Variables	Availability	
	Pearson chi-square	
	Degree of freedom	*P* value
Frequency of supervision	3	0.003
Means of transportation cost coverage	3	0.018
Order change at a resupply point	1	0.017
Stockout at a resupply point	1	0.001

## Data Availability

The data used to support the findings of this study are available from the corresponding author upon request.

## Abbreviations

3TC: Lamivudine
ABC+3TC: Abacavir+lamivudine
AL: Artemether/lumefantrine
ART: Antiretroviral therapy
AZT+3TC: Zidovudine+lamivudine
AZT+3TC+NEV: Zidovudine+lamivudine+nevirapine
EMS: Essential medicines
EVF: Efavirenz
GAPPD: Global Action Plan for Pneumonia and Diarrhoea
HIV: Human immunodeficiency virus
IFRR: Internal facility report and requisition form
IV: Intravenous
LIAT: Logistics Indicators Assessment Tool
LPV/r: Lopinavir/ritonavir
MDG: Millennium Development Goal
NSAIDS: Nonsteroidal anti-inflammatory drugs
NVP: Nevirapine
ORS: Oral rehydration salt
ORT: Oral rehydration therapy
PFSA: Pharmaceuticals Fund and Supply Agency
SPSS: Statistical Package for the Social Sciences
UN: United Nation
UNFPA: United Nations Population Fund
WHO: World Health Organization.
